# Comparison of *de novo* assembly using long-read shotgun metagenomic sequencing of viruses in fecal and serum samples from marine mammals

**DOI:** 10.3389/fmicb.2023.1248323

**Published:** 2023-09-22

**Authors:** Katie Vigil, Tiong Gim Aw

**Affiliations:** Department of Environmental Health Sciences, School of Public Health and Tropical Medicine, Tulane University, New Orleans, LA, United States

**Keywords:** nanopore sequencing, metagenomics, viruses, marine mammals, bioinformatics

## Abstract

**Introduction:**

Viral diseases of marine mammals are difficult to study, and this has led to a limited knowledge on emerging known and unknown viruses which are ongoing threats to animal health. Viruses are the leading cause of infectious disease-induced mass mortality events among marine mammals.

**Methods:**

In this study, we performed viral metagenomics in stool and serum samples from California sea lions (*Zalophus californianus*) and bottlenose dolphins (*Tursiops truncates*) using long-read nanopore sequencing. Two widely used long-read de novo assemblers, Canu and Metaflye, were evaluated to assemble viral metagenomic sequencing reads from marine mammals.

**Results:**

Both Metaflye and Canu assembled similar viral contigs of vertebrates, such as *Parvoviridae*, and *Poxviridae*. Metaflye assembled viral contigs that aligned with one viral family that was not reproduced by Canu, while Canu assembled viral contigs that aligned with seven viral families that was not reproduced by Metaflye. Only Canu assembled viral contigs from dolphin and sea lion fecal samples that matched both protein and nucleotide RefSeq viral databases using BLASTx and BLASTn for *Anelloviridae*, *Parvoviridae* and *Circoviridae* families. Viral contigs assembled with Canu aligned with torque teno viruses and anelloviruses from vertebrate hosts. Viruses associated with invertebrate hosts including densoviruses, *Ambidensovirus*, and various *Circoviridae* isolates were also aligned. Some of the invertebrate and vertebrate viruses reported here are known to potentially cause mortality events and/or disease in different seals, sea stars, fish, and bivalve species.

**Discussion:**

Canu performed better by producing the most viral contigs as compared to Metaflye with assemblies aligning to both protein and nucleotide databases. This study suggests that marine mammals can be used as important sentinels to surveil marine viruses that can potentially cause diseases in vertebrate and invertebrate hosts.

## 1. Introduction

Worldwide, viruses are reported to cause 72% of the infectious disease-induced mass mortality events (ID MME) in marine mammals from 1955 to 2018, specifically morbilliviruses and *Influenza A* viruses (Sanderson and Alexander, 2020). The U.S. National Oceanic and Atmospheric Administration (NOAA) reports that around 49% of marine mammal unusual mortality events (UME) from 1991 to 2021 are classified as undetermined (Onens et al., 2023). Marine mammals infected with viruses may be more susceptible to other oceanic algal toxins and harmful bacteria such as *Vibrio* and *Klebsiella* exacerbated by climate change (Bogomolni et al., 2016; Siebert et al., 2017; Sanderson and Alexander, 2020). For example, increased water temperatures enhance the survival of *Vibrio parahaemolyticus* in marine environments, which was documented to cause die offs of northern sea otters in Alaska due to septicemia and enteritis (Burek et al., 2008). Furthermore, *Klebsiella pneumoniae* was introduced into the New Zealand sea lion population in 1998 and is known to cause endemic pup mortality (Castinel et al., 2007; Roe et al., 2015). Harmful algal blooms that produce brevotoxins (Flewelling et al., 2005), domoic acid (Lefebvre et al., 1999; Gulland, 2000; Lefebvre et al., 2016), and saxitoxin (Lefebvre et al., 2016; Fire et al., 2020) are documented to cause mortality in marine mammals and are becoming more prevalent globally due to climate change (Hendrix et al., 2021).

The monitoring of viruses in ocean ecosystems can increase the likelihood of detecting emerging infectious diseases. *Calicivirus* is a prime example of a zoonotic virus, with ocean origin, that spilled over from sea lion to swine, and is known to cause vesicular disease in marine mammals (Neill et al., 1995; Smith et al., 1998). Furthermore, UME first responders were documented to contract sealpox from marine mammals infected with *Parapoxvirus, with* symptoms presented as contagious pustular dermatitis or lesion (Clark et al., 2005; Roess et al., 2011). In 1988, a phocine distemper *Morbillivirus* caused the biggest ID MME, with over 18,000 harbor seals (*Phoca vitulina*) stranded in Europe (Dietz et al., 1989). Morbilliviruses have caused more than half of the ID MME’s in marine mammals since 1955 (Sanderson and Alexander, 2020) with symptoms of skin lesions, pneumonia, brain infections and pup abortions (Pomeroy et al., 2005; Duignan et al., 2014). Although distemper viruses are not readily transmissible to humans, it was documented that canine distemper virus can adapt to use human cell receptors suggesting potential zoonotic spillover (Bieringer et al., 2013; Sakai et al., 2013). *Influenza A virus* (IAV) is the second leading cause of viral ID MME’s in marine mammals after being reported in harbor seal die offs since 1979, causing acute hemorrhagic pneumonia (Webster et al., 1981b; Sanderson and Alexander, 2020). Several cases of conjunctivitis caused by an IAV virus spillover event from seals to humans were documented in 1981 (Webster et al., 1981a). Therefore, there is a critical need to develop more comprehensive, rapid, and affordable detection methods for zoonotic viruses in marine environments to prevent the spread of emerging and endemic infectious diseases.

Viral diversity in marine environments is immense, and most viruses cannot be identified using traditional culturing techniques (Noble and Fuhrman, 1997; Munang'andu et al., 2017; Arya, 2020). Early metagenomics studies revealed that most of the viral diversity remains uncharacterized (Breitbart et al., 2002). Next generation sequencing (NGS) technologies combined with reliable bioinformatic pipelines are critical for the detection and characterization of novel and existing viral pathogens in marine mammals that could cross over to other vertebrate populations. Although the second generation short-read (200–400 bp) technologies, such as Illumina or Ion Torrent have high read throughput, the shorter DNA fragments can be difficult to assemble and annotate (Pop and Salzberg, 2008; Hu et al., 2021). The third generation nanopore sequencing technology is providing a new opportunity to develop more rapid, portable, and cost-effective genomic sequencing assays for viruses. For example, the nanopore-based sequencer MinION can produce read lengths of over 10 kB, which can overcome annotation of genomic repeat regions and structural variations that are difficult to assemble (Tørresen et al., 2019; Hu et al., 2021). Nanopore sequencing has been used to detect various viruses from diverse clinical samples (Quick et al., 2017; Hayashida et al., 2019; Cohen et al., 2020; Brown et al., 2021). This technology has been shown to sequence the full genome of four variants of herpes simplex viruses in single read, with read lengths ranging from 100 kb to 2.3 Mb (Saranathan et al., 2022). To our knowledge, the nanopore long-read sequencing technology has not been evaluated for non-targeted metagenomic sequencing of viruses in marine mammals.

*De novo* assemblers are programs that assemble shorter nucleotide sequences into longer fragments called contigs without a reference database. Many *de novo* assemblers that exist for long-read sequencing technologies have been evaluated only using bacteria (Chin et al., 2013; Zimin et al., 2013; Kamath et al., 2017; Koren et al., 2017; Kolmogorov et al., 2020), plants (Chin et al., 2016; Ruan and Li, 2020), and fungi (Chin et al., 2016) samples. Choosing the right *de novo* assembler is important for constructing error-free and artifact-less genome assemblies. *De novo* assembly algorithms include overlap-layout-consensus (OLC), de-Bruijn-graph (DBG), string-graph (SG) and hybrid approaches (Dida and Yi, 2021). Briefly, the OLC algorithm finds overlaps between reads, creates a read layout, then a consensus sequence is produced (Idury and Waterman, 1995; Li et al., 2012; Liao et al., 2019). DBG is an algorithm that chops reads up into short k-mers (substrings of length *k*), where overlapping edges (*k*−1) are found, resulting in an Eulerian (edges) or Hamiltonian (nodes) path to create a graph (Pevzner et al., 2001; Li et al., 2012) where contigs are constructed (Compeau et al., 2011). SG is a simplified OLC where sequence reads (nodes) and non-transitive edges produce suffix to prefix overlaps (Liao et al., 2019). Canu (Koren et al., 2017) is an upgraded long-read OLC assembler algorithm that integrates newer computational procedures to overcome noisy overlapping reads and decreases assembly time compared to the now unsupported Celera Assembler (Myers et al., 2000; Miller et al., 2008). Metaflye is a long-read DBG assembler algorithm that constructs repeat graphs from arbitrary paths called disjoints that are stringed together to construct contigs (Kolmogorov et al., 2019, 2020). Both Metaflye and Canu have been evaluated to construct genomes of plant (*Arabidopsis thaliana*), bacteria (*Escherichia coli, Bacillus cereus,* and *Staphylococcus aureus*), human, and yeast (*Saccharomyces cerevisiae*) (Dida and Yi, 2021). Metaflye outperformed Canu by generating larger contigs and higher N50 values, but is prone to more mis-assemblies and mismatches (Dida and Yi, 2021).

The objectives of this study were to (i) use metagenomics to characterize viruses in stool and serum samples from bottlenose dolphins (*Tursiops truncates*) and California sea lions (*Zalophus californiansus*) with a long-read nanopore sequencing approach; (ii) compare two *de novo* assemblers, Canu v2.2 and Metaflye v2.9.1, in generating viral contigs for annotation. The improved knowledge on marine viruses and successful protocol development will lead to a translational science that informs protection of animals and public health under a One Health framework.

## 2. Materials and methods

### 2.1. Sample collection

Five sea lion fecal, four sea lion serum, four dolphin fecal, and four dolphin serum samples were collected from the U.S. Navy’s dolphin and sea lion clinic facility in Point Loma, CA (Lat 32.746021, Long −117.237030) in 2018 and 2019, respectively. Samples were collected in 10 mL conical tubes and frozen at –80°C until analysis.

Samples from Navy animals were collected during their routine care and under the authority codified in U.S. Code, Title 10, Section 7524. Secretary of Navy Instruction 3900.41H directs that Navy marine mammals be provided the highest quality of care. The U.S. Navy Marine Mammal Program (MMP), Naval Information Warfare Center (NIWC) Pacific, houses, and cares for a population of bottlenose dolphins and California sea lions in San Diego Bay (CA, United States). NIWC Pacific is accredited by The Association for Assessment and Accreditation of Laboratory Animal Care (AAALAC) International and adheres to the national standards of the U.S. Public Health Service policy on the Humane Care and Use of Laboratory Animals and the Animal Welfare Act.

### 2.2. Samples processing for viral metagenomics

Animal fecal samples were weighed in 50 mL sterile conical tubes. Sterile phosphate-buffered saline (PBS) was added at 1 mL/g and vortexed for 5 min. Sterile PBS was also used as a negative control. The samples were centrifuged at 15,000*g* for 10 min, and the supernatant was filtered through 0.45 μm and 0.22 μm filters. Serum samples were filtered directly through 0.45 μm and 0.22 μm filters. In order to remove any free DNA, 1 mL aliquot of each sample was incubated with Ambion™ DNase I (RNase Free) (Thermo Fisher Scientific, United States) at 37°C for 1 h in a water bath at a final concentration of 0.1 U (unit) per μL. The DNase was deactivated by treating with 50 mM EDTA at 75°C in a heat block for 10 min.

### 2.3. Nucleic acid extraction

Viral nucleic acids were extracted using the Invitrogen™ PureLink™ Viral RNA/DNA Extraction kit according to manufacturer’s instructions (Thermo Fisher Scientific, United States). In this study, 200 μL of pre-treated samples were extracted and eluted into 50 μL sterile RNase-free water. Nucleic acid samples were stored in −80°C until further processing.

### 2.4. Random PCR (rPCR) assay

To generate sufficient material for sequencing, a random reverse transcription/amplification protocol was used to amplify both viral DNA and RNA. Briefly, 5 μL of the nucleic acid samples were mixed with 1uL of primer-A (5’-GTTTCCCAGTCACGATCNNNNNNNNN) (40 μM) (Wang et al., 2002, 2003), 4 μL nuclease-free water, and was heated to 65°C for 5 min and 22°C for 5 min. The 10 μL reaction was then added to 4 μL 5X First Strand Buffer [250 mM Tris–HCl (pH 8.3), 375 mM KCl, 15 mM MgCl_2_], 0.4 μL deoxynucleotide Solution Mix (dNTP) (10 mM) (New England Biolabs, United States), 0.6 μL nuclease-free water, 2 μL DTT (100 mM), 2uL SuperScript™ III Reverse Transcriptase (200 U/uL) (Thermo Fisher Scientific, United States), 1uL RNaseOUT™ (40 U/uL) (Thermo Fisher Scientific, United States) and was heated to 42°C for 60 min, 94°C for 2 min, and 10°C for 5 min. The 20 μL reaction was added to 2 μL 5X Sequenase™ Reaction Buffer, 7.7 μL nuclease-free water, 0.3 μL Sequenase™ Version 2.2 DNA Polymerase (13 U/μL) (Thermo Fisher Scientific, United States), and was heated to 10°C for 5 min, 37°C for 8 min with 1°C/s ramping, 94°C for 2 min, 10°C for 5 min (1.2 μL of 2.5 μL Sequenase™ Version 2.2 DNA Polymerase (13 U/uL) with 7.5 μL Sequenase™ Enzyme Dilution Buffer was added to each tube during this step), 37°C for 8 min with 1°C/s ramping, and 94°C for 8 min. 6 μL of the cDNA template was added to 8 μL MgCl_2_, 10 μL Buffer II (100 mM Tris–HCl, pH 8.3, 500 mM KCl), 1 μL dNTP (10 mM), 1 μL Primer-B (5’-GTTTCCCAGTCACGATC) (100 μM) (Wang et al., 2002, 2003), 1 μL AmpliTaq Gold™ DNA Polymerase with Gold Buffer and MgCL_2_ (Thermo Fisher Scientific, United States), and 73 μL nuclease-free water. The cDNA was amplified under the following conditions, 94°C for 15 min, 40 cycles of 94°C for 30 s, 40°C for 30 s, 50°C for 30 s, and 72°C for 1 min. The resulting PCR product was run on a 1% agarose gel and a smear between 500 bp to 1 kb was considered positive for sequencing. Positive samples were purified using the Wizard SV Gel and PCR Clean-Up System according to manufacturer’s instructions (Promega, Madison, WI, United States), where 100 μL cDNA was purified and eluted into 50 μL nuclease-free water. Samples were not normalized to a specific concentration prior to barcoding for sequencing.

### 2.5. Virome library preparation and nanopore sequencing

Amplified samples were checked for DNA concentration using Qubit^®^ 4 dsDNA HS Assay kit according to manufacturer’s instructions (Thermo Fisher Scientific, United States), and 1 μL samples were tested for concentration (ng/μL). 50 μL of purified rPCR samples were barcoded using the Oxford Nanopore PCR Barcoding Kit (SQK-PBK004) Version: RPB_9059_v1_revN_14Aug2019 according to the manufacturer’s instructions (Oxford Nanopore, United Kingdom). All samples were sequenced on the flow cell MinION Mk1b with R9.4.1 flow cell chemistry. Due to the frequent software updates, different version of MinKNOW and Guppy basecaller were used (MinKNOW v20.10.3 and v22.05.5 with Guppy basecaller v4.4.1 and v6.1.7 for fecal samples; MinKNOW v21.02.1 with Guppy basecaller v4.3.4 for all serum samples). The libraries were analyzed in the MinKNOW software under the following parameters: PCR Barcoding kit (SQK-PBK004), Native Barcoding Expansion 1–12 (EXP-PBC001), fast basecalling, trim barcodes, mid-read barcode filtering and barcode trimming. Fastq files were concatenated after sequencing for data analysis and bioinformatics.

### 2.6. Data analysis and bioinformatics

**Figure 1:**
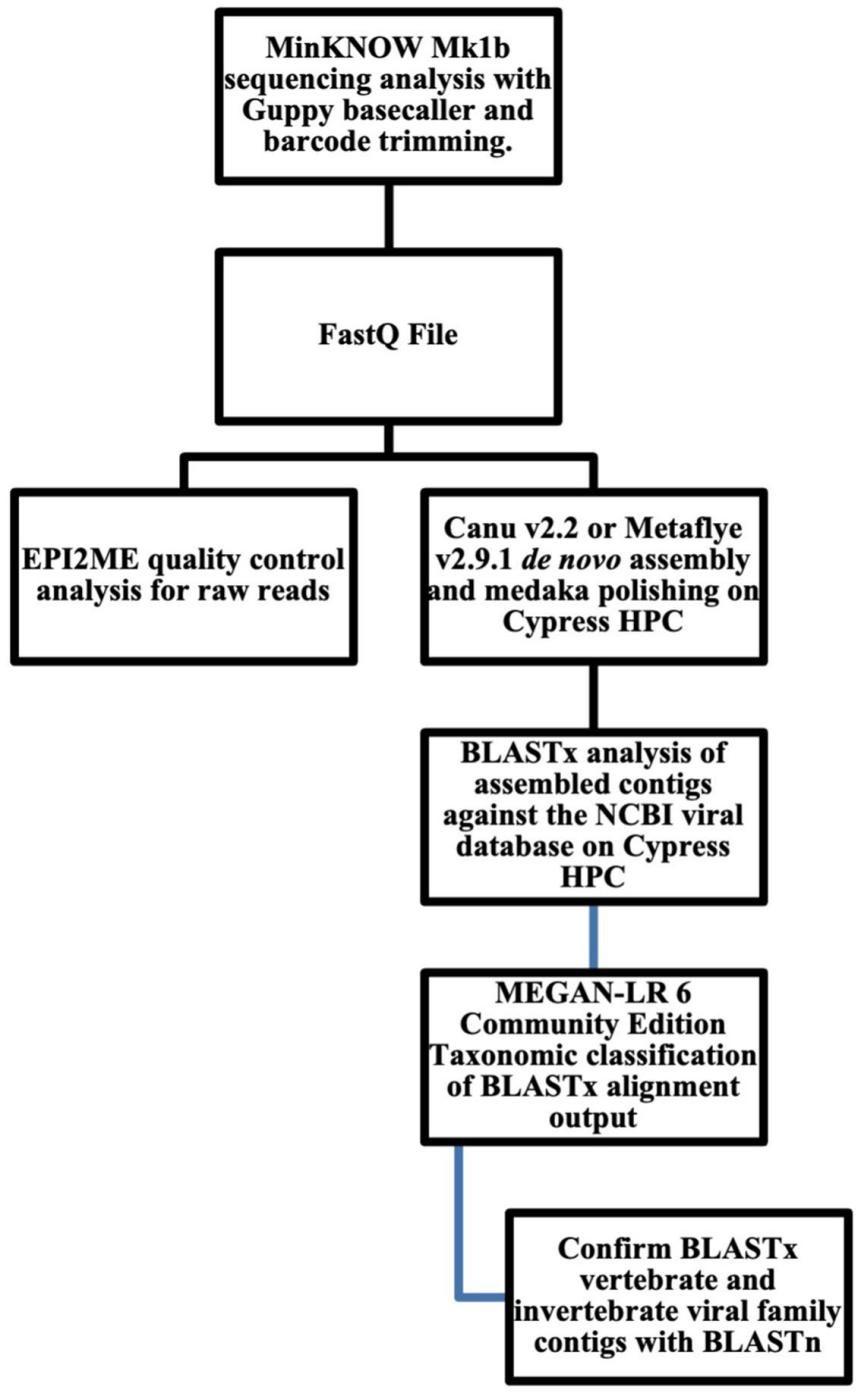
Bioinformatics workflow for long-read viral metagenomics.

The *in silico* pipeline analysis for nanopore sequencing data is shown in (Figure 1). Fastq files were uploaded to the Oxford Nanopore Technology (ONT) EPI2ME Software and analyzed using the “Whats in my pot?” WIMP (Humane + Viral) workflow to obtain quality control statistics on reads analyzed, total yield (Mb), average quality score, and average sequence length. Fastq sequencing files were analyzed in the Ubuntu 20.04 LTS 64-bit on Tulane Universities’ Cypress high performance computing (HPC) 124-node cluster with dual 10-core 2.8 GHz Intel Xeon E5-2680 v2 CPUs, 64 GB or RAM, and dual Xeon Phi 7120P coprocessor system. Fastq files were *de novo* assembled with Canu v2.2 and Metaflye v2.9.1 (Koren et al., 2017; Kolmogorov et al., 2020) pipeline and polished with Medaka v1.6.0^1^ (Lee et al., 2021). Canu 2.2 program parameters for low coverage reads were genomeSize = 2 m maxinputCoverage = 10,000 corOutCoverage = 10,000 corMhapSensitivity = high corMinCoverage = 0 redMemory = 32 oeaMemory = 32 batMemory = 64 minInputCoverage = 0 stopOnLowCoverage = 0, and genomeSize = 2 m maxInputCoverage = 100 –nanopore fecal samplesDNA.fastq for high coverage data, respectively. Metaflye v2.9.1 program parameters were –meta –threads 20 –nano-raw. Medaka v1.6.0 program parameters were medaka_consensus -i ${BASECALLS} -d ${DRAFT} -o ${OUTDIR} -t ${NPROC} -m r941_min_high_g330. All contigs were annotated using Basic Local Alignment Search Tool (BLAST) against the National Center for Biotechnology Information (NCBI) GenBank protein database (viral RefSeq). BLASTx hits with e-values ≤10^−4^ were used for analysis. BLASTx output files were analyze to viral families using MEGAN-LR (long-read) Community Edition 6.24.1 with default parameters, min score 50.0, max expected 0.0001, min percent identity 10.0, top percent 10.0, min support percent 0.01, min support 1, min read length 0, LCA algorithm longReads, percent to cover 80.0, and read assignment mode readCount. Viral family contigs that were positive for viral families that infect invertebrate and vertebrate hosts were subject to manual BLASTn analysis for confirmation. Version 5 BLAST+ 2.10.0 was used for both BLASTx and BLASTn database queries. The Quality Assessment Tool for Genome Assemblies (QUAST) v5.2 MetaQUAST was used to analyze contig output files. To determine if Canu v2.2^2^ assembles more viral family reads compared to MetaFlye v2.91,^3^ a one-tailed Wilcoxon rank-sum test (also known as the Mann–Whitney U test) for non-parametric data was used. Statistical analysis was performed using R version 4.2.3 (R Core Team, 2013).

The original nucleotide sequences described in this study have been deposited in the GenBank database under the Bioproject accession numbers PRJNA998092.

## 3. Results

### 3.1. Generation of viral metagenomic sequences

A long-read sequencing of randomly primed amplicons using the nanopore MinION generated a total of 1,698,981 sequencing reads, yielding 1,557 Mb after quality filtering of “passed” reads. The highest number of sequencing reads at 1,120,197 reads after basecalling, were produced from sea lion fecal samples, followed by dolphin fecal samples with 456,126 sequence reads after basecalling (Table 1). The average quality score of all the sequences ranged from 9 to 15, with the average sequence length (bases) ranging from 627 to 769 bases (Table 1).

**Table 1:** EPI2ME quality control analysis of passed raw read fastq files generated from the MinKNOW software.

Marine mammal	Number of samples	Sample type	Reads analyzed (bases)	Total yield (Mb)	Average quality score	Average sequence length (bases)
Sea lion	5	Stool	1,120,197	1,194	9	769
Sea lion	3	Serum	71,121	44	15	627
Dolphin	4	Stool	456,126	284	9	721
Dolphin	4	Serum	51,537	35	10	684

### 3.2. *De novo* assembly comparison

Canu v2.2 assembled a total of 333 contigs, with 118 viral contigs ranging from 1,029 to 13,513 nucleotides (nt) long for dolphin samples and 593 total contigs, with 224 viral contigs ranging from 1,026 to 8,114 nt for sea lion samples. In this study, viral contigs are the assembled sequences that aligned with the reference sequences from the NCBI Viral RefSeq database using BLASTx. Metaflye v2.9.1 assembled a total of 177 contigs, with 76 viral contigs ranging from 262 to 3,631 nt for dolphin samples and a total of 130 contigs, with 46 viral contigs ranging from 1,128 to 4,740 for sea lion samples (Table 2). Canu v2.2 produced the longest contig at 13,513 nt from dolphin fecal samples. Metaflye v2.9.1 produced higher N50 values compared to Canu v2.2 for both dolphin and sea lion fecal and serum samples. Canu v2.2 produced more contigs that could be annotated as viruses using the NCBI database compared to Metaflye v2.9.1. The mean viral contig size was higher for dolphin fecal samples, but lower in sea lion fecal samples with Canu v2.2 compared to Metaflye v2.9.1. The mean viral contig size was lower for dolphin serum samples, but higher for sea lion serum samples with Canu v2.2 compared to Metaflye v2.9.1. Metaflye v2.9.1 did not produce viral contigs for sea lion serum samples. Canu v2.2 produced lower percent viral contigs for dolphin fecal samples, but higher percent viral contigs for sea lion fecal samples, while Metaflye v2.9.1 produced higher percent viral contigs for dolphin serum. Overall, Canu v2.2 used lower central processing units (CPUs) compared to Metaflye v2.9.1 but took a longer time to assemble contigs compared to Metaflye v2.9.1 (Table 2).

**Table 2:** A comparison of the performance and annotation outputs for *de novo* assemblers Canu v2.2 and Metaflye v2.9.1.

Samples	Canu v2.2				Metaflye 2.9.1			
	Fecal samples		Serum samples		Fecal samples		Serum samples	
	Dolphin	Sea lion	Dolphin	Sea lion	Dolphin	Sea lion	Dolphin	Sea lion
Total contigs	290	544	63	103	175	128	2	2
Largest contig (nt)	13,513	8,114	1915	1967	3,631	4,740	2,501	2,670
N50 (nt)	1,198	1,194	1,173	1,164	2,318	2,302	2,499	2,658
Total non-viral contigs	162	280	53	89	101	82	0	2
Total viral contigs	108	213	10	11	74	46	2	0
Viral contig size range (nt)	1,029–13,513	1,026–8,114	1,055–1781	1,043–1,482	262–3,631	1,128–4,740	2,214–2,501	0
Mean viral contig size (nt)	2,346	1,670	1,346	1,278	2,185	2,363	2,358	0
Median viral contig size (nt)	1,744	1,375	1,317	1,272	2,298	2,349	2,358	0
% Viral contigs	37%	39%	16%	11%	42%	36%	100%	0%
GC (%)	41.96	41.19	47.69	47.14	41.41	41.18	43.86	55.28
Total viral BLASTx hits	982	1,377	113	51	758	207	20	0
No. of CPUs	10	10	10	10	20	40	20	20
Run time (min)	1,460	1980	270	330	60	15	5	4

BLASTx outputs with MEGAN were used to determine viral contigs, which are contigs that aligned against the NCBI RefSeq viral database.

### 3.3. The distribution of viral contigs

**Figure 2:**
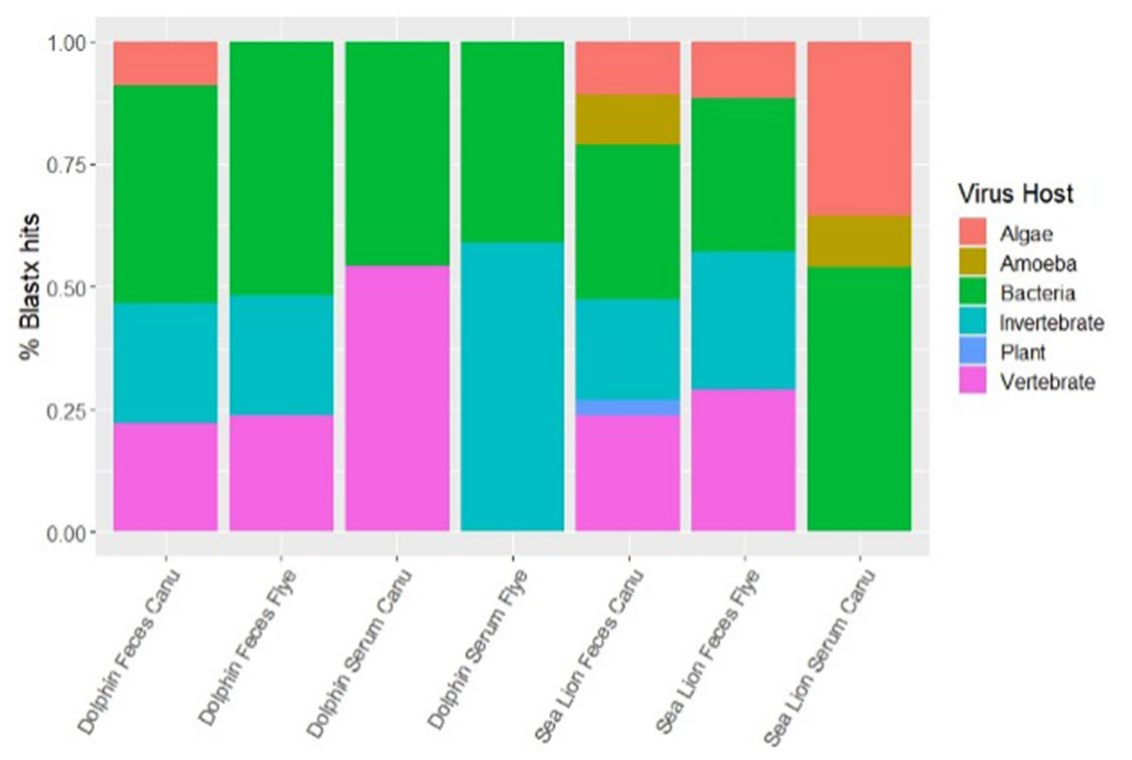
Percent distribution of contigs by types of viral host in dolphin and sea lion fecal and serum samples. Contigs were assembled using Canu v2.2 and Metaflye v2.9.1 and annotated using BLASTx program against the NCBI Virus database.

**Figure 3:**
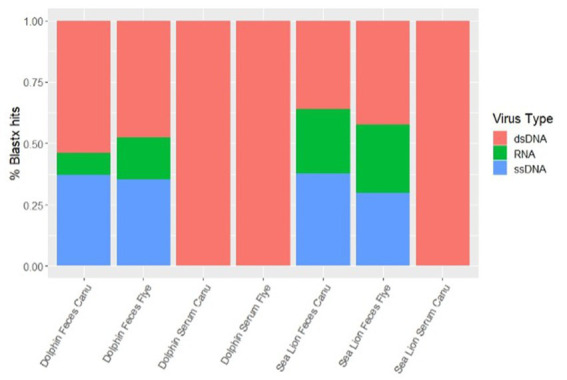
Percent distribution of contigs by types of viral genome in dolphin and sea lion fecal and serum samples. Contigs were assembled using Canu v2.2 and Metaflye v2.9.1 and annotated using BLASTx program against the NCBI Virus database.

Using different *de novo* assemblers, contigs from diverse viral host distributions were observed. For dolphin fecal samples, both Metaflye v2.9.1 and Canu v2.2 assembled viral contigs that aligned with bacteriophages, invertebrate, and vertebrate viruses, but only Canu v2.2 produced contigs that aligned with algal viruses (Figure 2). Overall, Canu v2.2 produced viral contigs from more diverse hosts in fecal samples compared to Metaflye v2.9.1 (Figure 2). Vertebrate hosts were dominant in dolphin serum using Canu v2.2, while invertebrate hosts were dominant in dolphin serum using Metaflye v2.9.1. Contigs aligning with viruses from amoeba hosts were only detected in sea lion fecal and serum samples using Canu v2.2. Contigs aligning to plant viral hosts were only detected in sea lion fecal samples with Canu v2.2. Distribution of virus types between samples and *de novo* assemblers were similar. All serum samples had contigs that aligned 100% with dsDNA type viruses. Both dolphin and sea lion fecal samples had contigs that aligned with dsDNA, RNA and ssDNA virus types using either Canu v2.2 or Metaflye v2.9.1. Contigs that aligned with RNA viruses had slightly higher distribution in sea lion fecal samples compared to dolphin fecal samples, while ssDNA types had similar distribution (Figure 3).

**Figure 4:**
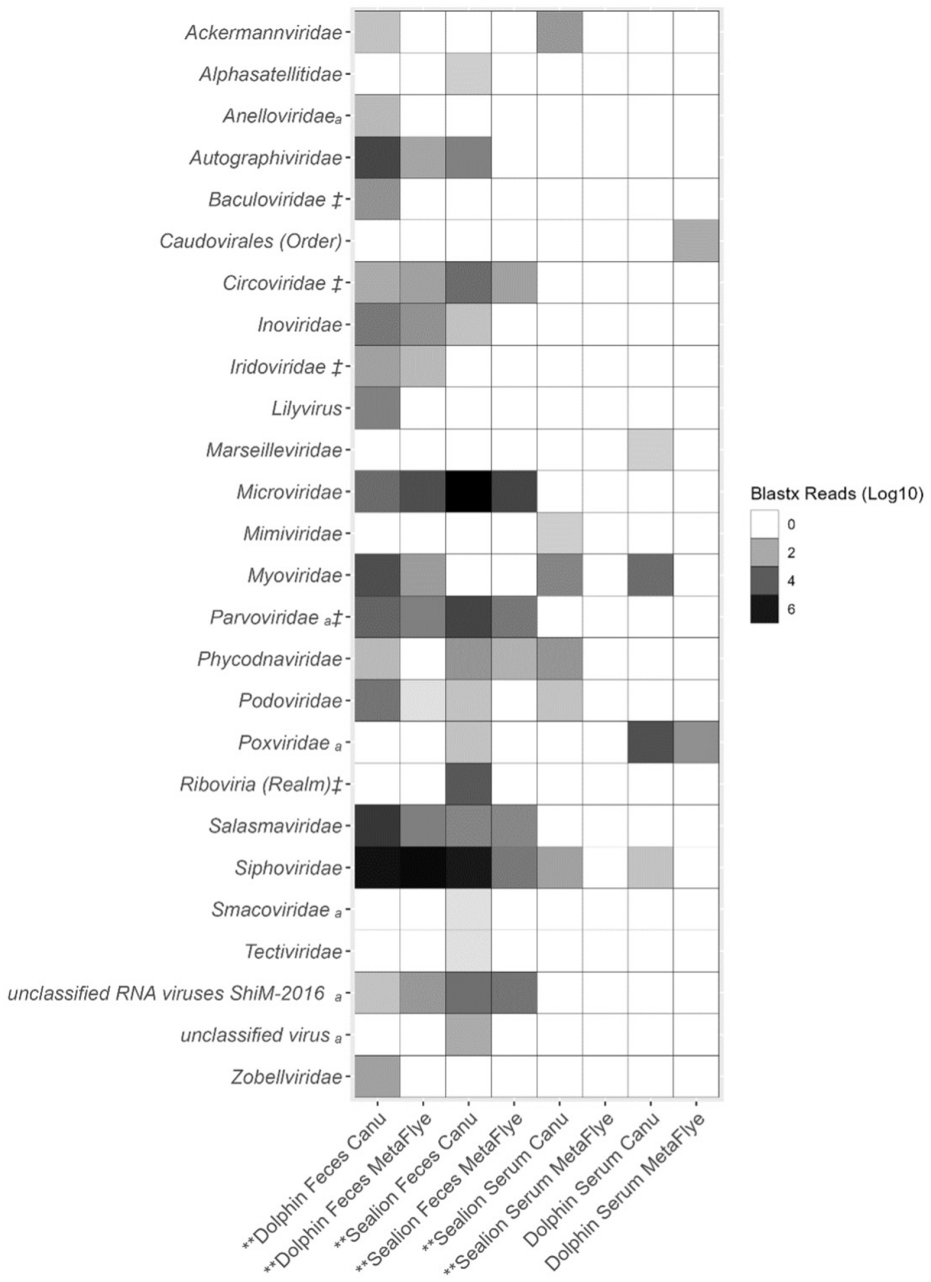
Total viral family reads from BLASTx (e-value = ≤10^−4^) output files of assembled contigs using Canu v2.2 and Metaflye v2.9.1 against the NCBI Virus Refseq database from dolphin and sea lion fecal samples and serum. ^a^vertebrate viral hosts and ‡ invertebrate viral hosts. Asterisks (**) represent *p*-value = <0.05 for the one-tailed Wilcoxon Rank Sum test for non-parametric log10 data specifying that the alternative hypothesis is that Canu v2.2 assembled viral reads were significantly greater than MetaFlye v2.9.1.

Canu v2.2 assembled significantly higher numbers of viral contigs compared to Metaflye v2.9.1 for dolphin fecal samples, sea lion fecal samples and sea lion serum samples (*p*-value = <0.05) (Figure 4). Assembled viral contigs aligned with several vertebrate viral families such as *Anelloviridae, Parvoviridae, Poxviridae, Smacoviridae* using the protein BLASTx program (Figure 4). Invertebrate viruses detected in fecal samples of dolphin and sea lion aligned to the families of *Baculoviridae, Circoviridae, Iridoviridae,* and *Parvoviridae*. *Baculoviridae* viral reads only aligned with Canu v2.2 assembled viral contigs from dolphin fecal samples. *Circoviridae, Parvoviridae, Riboviria* (realm) viral reads were higher in sea lion fecal samples with Canu v2.2.

Bacteriophage families include *Autographiviridae, Inoviridae, Myoviridae, Podoviridae, Salasmaviridae, Siphoviridae, and Zobellviridae* were also detected in dolphin fecal samples using Canu v2.2 (Figure 4). In sea lion fecal samples, bacteriophage families included *Microviridae* and *Tectiviridae* were detected.

**Figure 5:**
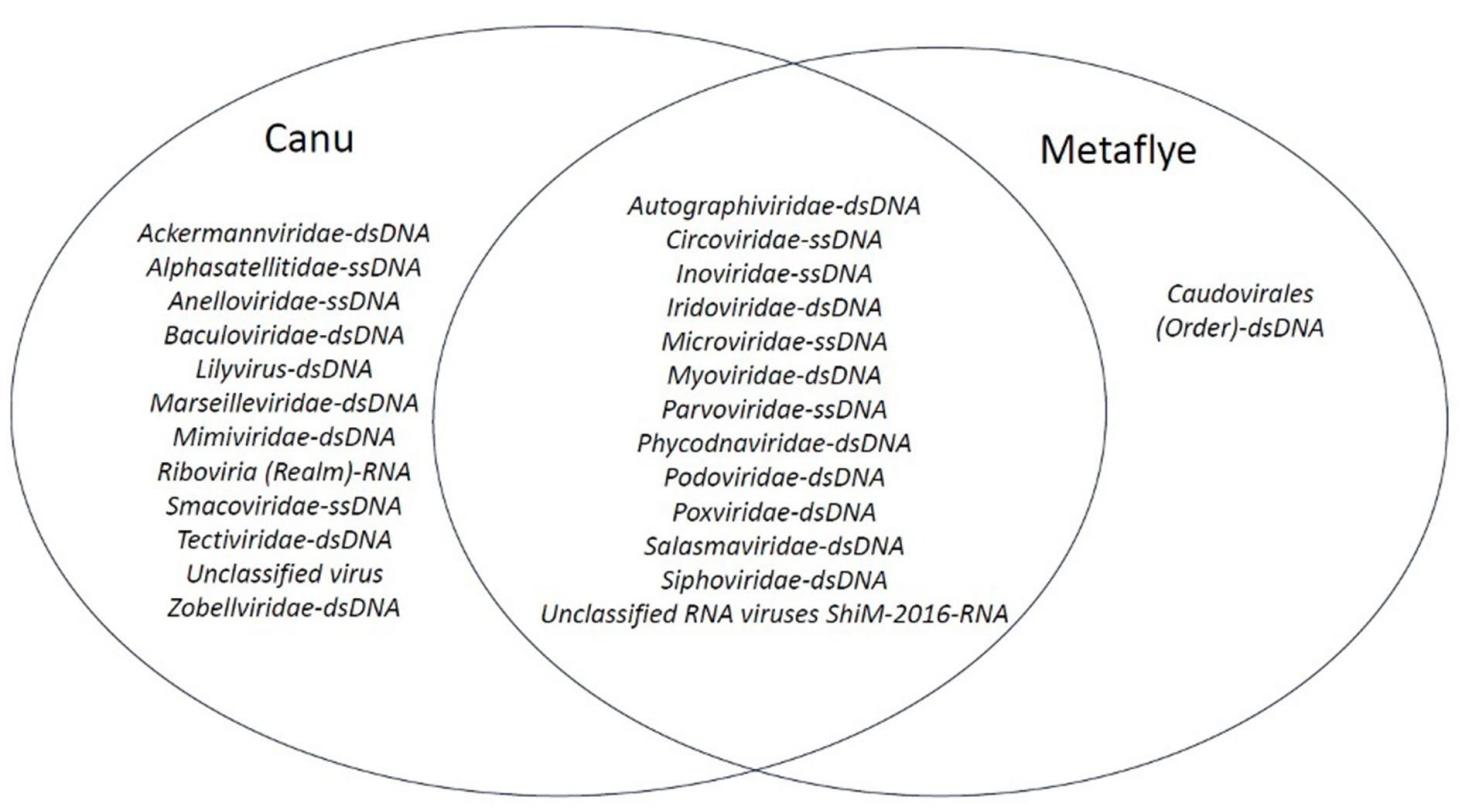
Venn diagram of viral families that aligned with contigs assembled by Canu v2.2 and Metaflye v2.9.1 from BLASTx (e-value = ≤10^−4^) output files against the NCBI Virus Refseq database.

Although Canu v2.2 and Metaflye v2.9.1 assembled viral contigs from similar viral families, some discrepancies were found. Canu v2.2 produced viral contigs of seven viral families that were absent from the Metaflye v2.9.1 output (Figure 5). Overall, most of the viruses that were detected with both Canu v2.2 and Metaflye v2.9.1 were linear, non-enveloped dsDNA viruses (Supplementary Data_Sheet_1_virus_genome excel file).

### 3.4. Protein and nucleotide analysis

Viral contigs that were aligned using the protein (amino acid, aa) BLASTx program were further confirmed using the nucleotide BLASTn program. Canu v2.2 generated contigs of vertebrate and invertebrate viruses that were positive for both protein and nucleotide NCBI RefSeq viral databases, while Metaflye v2.9.1 only generated viral contigs that had positive viral families for protein, not nucleotide. No viral contigs from vertebrate and invertebrate hosts were identified in serum samples using both assemblers with both BLASTx and BLASTn searches. Dolphin fecal samples contained viral contigs that were found to be associated with seals, sea stars, and oysters, as confirmed by both protein and nucleotide databases. These viruses included torque teno midi virus (TTMDV), seal annellovirus, and sea star densoviruses (DNV) (Table 3). Sea lion fecal samples contained viral contigs that were also detected in oysters, fish, sea stars, crayfish, and clams. These viruses included sea star DNV, *Cherax quadricarinatus* DNV, *Circoviridae* species., and *Ambidensovirus* (AmDNV) (Table 4).

**Table 3:** Canu v2.2 assembled vertebrate and invertebrate viral contigs from pooled dolphin fecal samples (*n* = 4) that shared the same viral families between BLASTx and BLASTn.

Blast type	Viral family	Source taxonomy	Source name	Contig size (nt)	Viral assignment	NCBI accession #	ID%	e-value	Bit score
BLASTx	*Anelloviridae*	*Homo sapiens*	Human	1,257	Torque teno midi virus 12	YP_009505786.1	32	3.00E-07	51
	*Anelloviridae*	*Phoca vitulina*	Harbor seal	1,257	Seal anellovirus 4	YP_009115496.1	33	4.00E-11	54
	*Anelloviridae*	*Phoca vitulina*	Harbor seal	1,257	Seal anellovirus 4	YP_009115496.1	33	8.00E-06	51
	*Anelloviridae*	*Ailurus fulgens*	Red panda	1,257	Lesser panda anellovirus	YP_009551687.1	38	2.00E-10	56
	*Parvoviridae*	*Asterias amurensis*	N.Pacific seastar	1,625	Sea star-associated *densovirus*	YP_009507339.1	59	1.00E-04	223
	*Parvoviridae*	*Asterias amurensis*	N.Pacific seastar	1,625	Sea star-associated *densovirus*	YP_009507339.1	46	4.00E-16	85
	*Parvoviridae*	*Asterias amurensis*	N.Pacific seastar	2,929	Sea star-associated *densovirus*	YP_009507339.1	46	1.00E-04	602
BLASTn	*Anelloviridae*	*Arctocephalus australis*	So. American fur seal	1,257	Torque teno *arctocephalus australis* virus 1	MW504281.1	79	6.46E-148	536
	*Anelloviridae*	*Arctocephalus australis*	So. American fur seal	1,257	Anellovirus fur seal/AAUST60/BR/2012	MW504281.1	84	2.71E-37	180
	*Parvoviridae*	*Crassostrea ariakensis*	Suminoe oyster	1,625	*Ambidensovirus*	KY548840.1	76.73	5.00E-140	510
	*Parvoviridae*	*Asterias forbesi*	Forbes sea star	1,625	Uncultured *densovirus*	MN190158.1	82.23	2.00E-70	345
	*Parvoviridae*	*Pisaster ochraceus*	Purple sea star	2,929	Uncultured *densovirus*	MW073776.1	81.85	1.33E-63	257
	*Parvoviridae*	*Asterias forbesi*	Forbes sea star	2,929	Uncultured *densovirus*	MN190158.1	79.29	8.02E-61	248
	*Parvoviridae*	*Asterias amurensis*	N.Pacific seastar	2,929	Sea star-associated *densovirus*	NC_038532.1	79.13	8.13E-51	215
	*Parvoviridae*	*Asterias forbesi*	Forbes sea star	2,929	Sea star-associated *densovirus*	KY785181.1	79.79	4.89E-48	206
	*Parvoviridae*	*Asterias forbesi*	Forbes sea star	2,929	Sea star-associated *densovirus*	KY785180.1	79.44	2.28E-46	200
	*Parvoviridae*	*Yangtze River*	N/A	2,929	*Parvoviridae* sp. Isolate	MW348572.1	78.37	3.86E-34	159
	*Parvoviridae*	*Yangtze River*	N/A	2,929	*Parvoviridae* sp. Isolate	MW348571.1	83.85	1.41E-23	124
	*Parvoviridae*	*Pycnopodia helianthoides*	Sunflower sea star	2,929	Uncultured *densovirus*	MT733051.1	79.25	8.13E-51	215

The contigs with the same size (nucleotide, nt) were the same contigs, but resulted in multiple alignments from different taxonomic sources.

**Table 4:** Canu v2.2 assembled vertebrate and invertebrate viral contigs from pooled sea lion fecal samples (*n* = 5) that shared the same viral families between BLASTx and BLASTn.

Blast type	Viral family	Source taxonomy	Source name	Contig size (nt)	Viral assignment	NCBI accession #	ID%	e-value	Bit score
BLASTx	*Parvoviridae*	*Asterias amurensis*	N.Pacific seastar	2,303	Sea star-associated *densovirus*	YP_009507339.1	51	1.00E-04	286
	*Parvoviridae*	*Cherax quadricarinatus*	Australian red claw crayfish	2,303	*Cherax quadricarinatus densovirus*	YP_009134734.1	46.3	3.00E-34	144
	*Parvoviridae*	*Asterias amurensis*	N.Pacific seastar	2,309	Sea star-associated *densovirus*	YP_009507339.1	45.6	1.00E-04	347
	*Parvoviridae*	*Cherax quadricarinatus*	Australian red claw crayfish	2,309	*Cherax quadricarinatus densovirus*	YP_009134734.1	40.5	1.00E-04	345
	*Parvoviridae*	*Asterias amurensis*	N.Pacific seastar	2,309	Sea star-associated *densovirus*	YP_009507340.1	83.9	1.00E-04	251
	*Parvoviridae*	*Cherax quadricarinatus*	Australian red claw crayfish	2,309	*Cherax quadricarinatus densovirus*	YP_009134732.1	79.6	1.00E-04	234
	*Circoviridae*	*Paphies subtriangulata*	Clam	1,254	Avon-Heathcote Estuary associated circular virus 28	YP_009126886.1	40.1	6.00E-16	119
	*Circoviridae*	*Paphies subtriangulata*	Clam	1,254	Avon-Heathcote Estuary associated circular virus 28	YP_009126887.1	35	1.00E-29	81
	*Parvoviridae*	*Cherax quadricarinatus*	Australian red claw crayfish	1,480	*Cherax quadricarinatus densovirus*	YP_009134732.1	44.2	1.00E-04	294
	*Parvoviridae*	*Asterias amurensis*	N.Pacific seastar	1,480	Sea star-associated *densovirus*	YP_009507340.1	45.6	1.00E-04	291
	*Parvoviridae*	*Cherax quadricarinatus*	Australian red claw crayfish	1,480	*Cherax quadricarinatus densovirus*	YP_009134732.1	67.8	1.00E-30	128
	*Parvoviridae*	*Asterias amurensis*	N.Pacific seastar	1,480	Sea star-associated *densovirus*	YP_009507340.1	71.6	1.00E-04	124
	*Parvoviridae*	*Cherax quadricarinatus*	Australian red claw crayfish	1,480	*Cherax quadricarinatus densovirus*	YP_009134732.1	53.8	1.00E-04	90
	*Parvoviridae*	*Asterias amurensis*	N.Pacific seastar	1,480	Sea star-associated *densovirus*	YP_009507340.1	53.9	1.00E-04	86
	*Parvoviridae*	*Cherax quadricarinatus*	Australian red claw crayfish	1,174	*Cherax quadricarinatus densovirus*	YP_009134731.1	35.3	1.00E-04	184
	*Parvoviridae*	*Cherax quadricarinatus*	Australian red claw crayfish	1,408	*Cherax quadricarinatus densovirus*	YP_009134732.1	59.9	1.00E-04	463
	*Parvoviridae*	*Asterias amurensis*	N.Pacific seastar	1,408	Sea star-associated *densovirus*	YP_009507340.1	56.5	1.00E-04	451
	*Parvoviridae*	*Asterias amurensis*	N.Pacific seastar	1,034	Sea star-associated *densovirus*	YP_009507339.1	53.3	1.00E-04	240
	*Parvoviridae*	*Cherax quadricarinatus*	Australian red claw crayfish	1,093	*Cherax quadricarinatus densovirus*	YP_009134732.1	62.2	1.00E-04	325
	*Parvoviridae*	*Asterias amurensis*	N.Pacific seastar	1,093	Sea star-associated *densovirus*	YP_009507340.1	59.3	1.00E-04	318
	*Parvoviridae*	*Cherax quadricarinatus*	Australian red claw crayfish	1,093	*Cherax quadricarinatus densovirus*	YP_009134732.1	77.2	1.00E-19	94
	*Parvoviridae*	*Asterias amurensis*	N.Pacific seastar	1,093	Sea star-associated *densovirus*	YP_009507340.1	78.9	3.00E-19	92
	*Parvoviridae*	*Solenopsis invicta*	Ants	1,093	*Solenopsis invicta densovirus*	YP_008766862.1	22.5	6.00E-11	67
	*Circoviridae*	Ocean Water	N/A	1,312	*Circoviridae* 2 LDMD-2013	YP_009109630.1	33.3	1.00E-04	194
BLASTn	*Parvoviridae*	*Crassostrea ariakensis*	Suminoe oyster	2,303	*Ambidensovirus*	KY548840.1	76.052	2.02E-140	512
	*Parvoviridae*	*Crassostrea ariakensis*	Suminoe oyster	2,309	*Ambidensovirus*	KY548840.1	86.874	5.75E-126	464
	*Circoviridae*	*Lutjanus campechanus*	Red snapper	1,254	*Circoviridae* sp. isolate	MH616634.1	92.511	1.00E-04	649
	*Circoviridae*	*Oncorhynchus mykiss*	Rainbow trout	1,254	*Circoviridae* sp. isolate	MH617160.1	92.188	8.01E-177	632
	*Circoviridae*	*Lutjanus campechanus*	Red snapper	1,254	*Circoviridae* sp. isolate	MH616871.1	92.255	1.73E-173	621
	*Parvoviridae*	*Cygnus olor*	Mute swan	1,480	*Ambidensovirus*	MW588057.1	83.898	5.48E-20	111
	*Parvoviridae*	*Astropecten polyacanthus*	Comb sea star	1,480	Uncultured *densovirus*	MT733013.1	79.762	5.60E-05	62.1
	*Parvoviridae*	*Crassostrea ariakensis*	Suminoe oyster	1,174	*Ambidensovirus*	KY548840.1	76.509	1.74E-123	455
	*Parvoviridae*	*Crassostrea ariakensis*	Suminoe oyster	1,408	*Ambidensovirus*	KY548840.1	87.886	1.00E-04	1,439
	*Parvoviridae*	*Yangtze River*	N/A	1,408	*Parvoviridae* sp. Isolate	MW348572.1	78.683	1.00E-04	749
	*Parvoviridae*	*Pisaster ochraceus*	Purple sea star	1,408	Densovirinae sp. isolate	MW073782.1	74.863	3.08E-32	152
	*Parvoviridae*	*Pisaster ochraceus*	Purple sea star	1,034	Uncultured *densovirus*	MT733042.1	77.181	2.02E-107	401
	*Parvoviridae*	*Pycnopodia helianthoides*	Sunflower sea star	1,034	Uncultured *densovirus*	MT733032.1	75.478	2.04E-97	368
	*Parvoviridae*	*Crassostrea ariakensis*	Suminoe oyster	1,093	*Ambidensovirus*	KY548840.1	87.929	1.00E-04	1,061
	*Parvoviridae*	*Yangtze River*	N/A	1,093	*Parvoviridae* sp. Isolate	MW348572.1	79.642	3.23E-175	627
	*Parvoviridae*	*Pyloric caeca*	Starfish	1,093	Uncultured *densovirus*	MN190158.1	73.899	1.31E-89	342
	*Parvoviridae*	*Pycnopodia helianthoides*	Sunflower sea star	1,093	Uncultured *densovirus*	MT733031.1	73.089	6.19E-78	303
	*Parvoviridae*	*Pisaster ochraceus*	Purple sea star	1,093	Uncultured *densovirus*	MT733037.1	73.011	2.88E-76	298
	*Parvoviridae*	*Pyloric caeca*	Starfish	1,093	Uncultured *densovirus*	MT733024.1	72.982	2.88E-76	298
	*Parvoviridae*	*Pisaster ochraceus*	Purple sea star	1,093	Uncultured *densovirus*	MT733041.1	75.362	5.25E-14	91.6
	*Circoviridae*	*Lutjanus campechanus*	Red snapper	1,312	*Circoviridae* sp. isolate	MH617401.1	97.162	1.00E-04	1784
	*Circoviridae*	*Lutjanus campechanus*	Red snapper	1,312	*Circoviridae* sp. isolate	MH617399.1	78.808	7.89E-16	99

The contigs with the same size (nucleotide, nt) were the same contigs, but resulted in multiple alignments from different taxonomic sources.

## 4. Discussion

### 4.1. Viral metagenomics using nanopore sequencing and *de novo* sequence assembly

Assembling reliable metagenomic sequencing data is critical to characterizing viral diversity in marine environments. *De novo* assembly is important because it allows researchers to construct genomes without the need for a reference genome or when reference genomes are not available. *De novo* assembly can also discover novel genes and genetic variants of viruses (Allen et al., 2011; Chiu, 2013; Carbo et al., 2020). Several nanopore-based sequencing bioinformatic tools have been developed to handle the long-read sequencing reads, but many lack performance testing on unknown viral metagenomic sequencing dataset (Li, 2016; Kamath et al., 2017; Koren et al., 2017; Wang et al., 2018; Kolmogorov et al., 2020; Ruan and Li, 2020). This work aimed at filling this gap by using viral metagenomics data generated from marine mammal fecal and serum specimens with the nanopore sequencing platform.

Long-read sequencing technologies, such as the MinION nanopore sequencer, have several advantages over short-read sequencing technologies, e.g., it is portable, it does not require large imaging equipment to detect DNA nucleotides, lower cost, it can be powered through a Universal Serial Bus (USB) port, and it can be used in the field (Kono and Arakawa, 2019). In addition, nanopore sequencing can sequence longer stretches of DNA (>500 bp) (Adewale, 2020), can pick up long repetitive sequences (Kono and Arakawa, 2019), does not require fragmentation, and can directly sequence RNA molecules (Garalde et al., 2018). Although short-read sequencing can produce more reads with shorter lengths (<300 bp) (Hu et al., 2021), long-read sequencing is capable of sequencing full viral genomes, thus making assembly less error prone (Kono and Arakawa, 2019). Our results showed variation in results between the two *de novo* assemblers, Canu and Metaflye. Although Metaflye was a faster assembler that generated higher N50, it produced less viral alignments against the NCBI RefSeq viral protein database using BLASTx as compared to Canu. A study comparing Canu and Metaflye using sequences of bacteria, mammal, plant and fungi revealed that Metaflye outcompeted Canu with larger N50 values, but high error rates when assembling full genomes against reference sequences (Dida and Yi, 2021). Other studies show Canu and Metaflye performing similarly (Jung et al., 2020; Wang et al., 2021). Recent genome assembly pipelines created for viruses are using Canu for preprocessing of reads before reference alignment (Roach et al., 2022; Yu et al., 2023), suggesting that this assembler could produce quality assemblies for metagenomics analysis. *De novo* algorithms that generate large contigs and high N50s are considered good quality for genome assembly, but sometimes the results are inaccurate and produce more mismatches (Dida and Yi, 2021), which could be why Metaflye produced mismatches between viral contigs aligned against protein and nucleotide sequence databases for invertebrate and vertebrate viral families.

Canu was the only assembler that produced contigs that matched the same viral families between the protein and nucleotide databases from marine mammal fecal samples. This suggests that Canu could assemble contigs that are more accurate for viral annotation from environmental samples as compared to Metaflye. Canu is an OLC algorithm based *de novo* assembler, and is documented to have less error rates compared to DBG assemblers for long-reads (Kono and Arakawa, 2019). Vertebrate and invertebrate viruses in fecal samples of *Parvoviridae, Anelloviridae,* and *Circoviridae* families were confirmed with Canu assembler for both protein and nucleotide BLAST searches. In this study, there was a discrepancy between protein BLASTx and nucleotide BLASTn results for serum samples. For example, all the invertebrate and vertebrate viral contigs in serum samples identified using BLASTx against the protein database aligned with bacteriophages when using BLASTn against the nucleotide database. This could be that the lowest amount of sequence reads were generated in serum samples and both Canu and Metaflye are more accurate with higher sequencing read counts. *De novo* assembly could be less accurate with low sequencing depth due to insufficient genome coverage and limited redundancy for genome regions. *De novo* assemblers rely on read overlapping for OLC (Koren et al., 2017) or DBG (Kolmogorov et al., 2020) graph construction for contig or scaffold assembly. A low sequencing depth may result in reduced overlapping information making it harder for the algorithms to accurately detect sequencing overlaps. In addition, low read count may produce shorter contigs resulting in a fragmented assembly that may not accurately represent true structure of the sequenced genome regions. As seen in this study, the shortest mean nucleotide contig lengths were observed in serum samples and this could cause discrepancy in results between the two BLAST search programs. A future study could include a mock viral community to establish a baseline for sequencing reads prior to *de novo* assembly to better understand the relationship between sequencing depth and *de novo* assembly for viral metagenomics.

### 4.2. The detection of invertebrate and vertebrate viruses in fecal and serum samples of marine mammals using metagenomics

In this study, several viral contigs from dolphin and sea lion fecal samples aligned with viruses that were also isolated from human, mammal, seals, sea stars, bivalves, fish, birds and crayfish. These viruses include annelloviruses (AV), torque teno viruses (TTV), circoviruses and densoviruses. Multiple taxonomic sources aligned with the same viral contigs, which indicates that some viruses may infect multiple hosts (Tables 3 4). These results suggest that marine mammals can be used as important sentinel species (Bossart, 2006, 2011) to monitor marine environments for viruses that may spillover to different organisms.

First discovered in a serum samples from Hepatitis B (HBV) and Hepatitis C (HCV) patients (Khudair et al., 2019), TTV are single-stranded circular DNA (ssDNA) viruses, with a ~ 3.8 kb genome size and are currently classified into the *Anelloviridae* family under the genus *Alphatorquevirus* (Lolomadze and Rebrikov, 2020). TTV are known to be a diverse viral group with over 20 genotypes and 40% viral genome heterogeneity with frequent recombination in the N22 region of the open reading frame (ORF) 1 gene (Manni et al., 2002; Hino and Miyata, 2007; Hsiao et al., 2021). The TTV-like mini virus (TTMV) was added to the TTV group in 2000 (Takahashi et al., 2000) and has a genome size of ~2.9 kb (Hino and Miyata, 2007). Torque teno midi virus (TTMDV) is generally considered a non-pathogenic virus, and is commonly found in the virome of human blood (Cebriá-Mendoza et al., 2021). TTV have also been proposed as a fecal viral indicator for monitoring water quality (Griffin et al., 2008; Hamza et al., 2011; Haramoto et al., 2018; Tavakoli Nick et al., 2019). Viral contigs detected in dolphin fecal samples aligned with TTV’s from human (32% aa identity), South American fur seal (*Arctocephalus australis*) (79%–84% nt identities), and harbor seal (*Phoca vitulina*) (33% aa identity) sources. The South American fur seal *torque teno arctecephalus australis virus 1* that aligned at 79% nt identity with the dolphin viral contig was detected in 2018 from seals found dead on the Rio Grande do Sul State shore in Brazil (Canova et al., 2021). The TTV that aligned with human could be indicative of a multi-host virus and possible sources from human wastewater or stormwater runoff. TTV are known to be highly resistant to wastewater treatment processes and are frequently detected in wastewater influent samples (Carducci et al., 2006; Haramoto et al., 2008; Plummer et al., 2014; Tavakoli Nick et al., 2019).

The *Circoviridae* family are circular ssDNA viruses with genome sizes of 1.7–2.1 kb and are composed of two genera *Circovirus and Cyclovirus* (Breitbart et al., 2017). Viral contigs from sea lion fecal samples aligned with viruses from the *Circoviridae* family of clams (*Paphies subtriangulata*) (35%–40.1% aa identity), red snappers (*Lutjanus campechanus*) (79%–97% nt identity) and rainbow trout (*Oncorhynchus mykiss*) (92% nt identity) sources. The Avon-Heathcote Estuary associated circular virus 28 from clams that aligned with a sea lion viral contig is from a group of single-stranded DNA (ssDNA) viruses encoding a replication-associated protein (Rep) (CRESS) viruses (Dayaram et al., 2015). *Circoviridae* species have unknown implications of disease but can cause infections in fish. In fish, circoviruses have been associated with skin and fin infections, called cauliflower disease (Doszpoly et al., 2014).

The *Parvoviridae* family are non-enveloped ssDNA viruses with linear genomes of 4–6 kb, and are split up into two subfamilies *Parvovirinae* and *Densovirinae* (Cotmore et al., 2019). The subfamily, *Densovirinae* (commonly referred to as densoviruses) infect insects and invertebrates, notably decapod crustaceans (shrimp and crayfish) (Jackson et al., 2020) Viral contigs from dolphin and sea lion fecal samples aligned with sea star associated *densovirus* (SSaDV). SSaDV is associated with sea star wasting disease (SSWD) that caused mass mortality events in >20 species of asteroids since 2013 from Alaska to Southern California (Hewson et al., 2014, 2018). SSaDV from dolphin fecal samples aligned with SSaDV from the North Pacific sea star (46%–59% aa identity; 79% nt identitiy). Viral contigs from sea lion fecal samples aligned with diverse densoviruses from comb sea star (*Astropcten polyacanthus*) (80% nt identity), purple sea star (*Piesaster ochraceus*) (77% nt identity), sunflower sea star (75% nt identity), and starfish (*Pyloric caeca*) (74% nt identity) sources. Sea stars are considered keystone species that play a role in regulating other species, such as bivalves, snails and other invertebrates in marine ecosystems (Paine, 1966; Collinge et al., 2008; Menge and Sanford, 2013). Viral contigs from sea lion fecal samples aligned with *Cherax quadricarinatus densovirus* (CqDV) from the Australian red claw crayfish (*Cherax quadricarinatus*) (35.3%–79.6% aa identity). CqDV has been linked to mortalities in red claw crayfish, which is a significant threat to the aquaculture industry (Saoud et al., 2013; Bochow et al., 2015). In addition, crayfish has an important ecological role in maintaining water quality (Chen et al., 2022), economic importance by providing food and jobs (McClain and Romaire, 2004), cultural importance with festivals (Gutierrez, 1998), and scientific importance for studying neurobiology, behavior and genetics (Huber et al., 2011; Jiang et al., 2014; Bacqué-Cazenave et al., 2017; D’Agnese et al., 2020). Viral contigs from dolphin fecal samples aligned with AmDNV from the Suminoe oyster (*Crassostrea ariakensis*) (76.7% nt identity). AmDNV was first isolated in 2017 from the Suminoe oysters (*Crassostrea ariakensis*) and shared similar amino acid identities with SSaDV (76–89% nt identity). But AmDNV has not been documented to cause mortality in oysters, instead most likely originated from the surrounding ocean ecosystem (Kang et al., 2017). Viral contigs from sea lion fecal samples aligned with AmDNV from Suminoe oysters (76%–88% nt identity) from Wuxi City, Jiangsu province, China (Kang et al., 2017). Another viral contig from sea lion fecal samples aligned with a AmDNV from a mute swan (*Cygnus olor*) (84% nt identity) and is not associated with disease in this bird species and more research is needed to understand the potential impact on bird health.

### 4.3. Study limitations

The use of filtration and DNase treatment could potentially reduce viral concentrations and diversity due to losses during these sample processing steps. DNase is an enzyme that breaks down extracellular “naked” or “free” DNA that are present in fecal and serum samples. In theory, intact viral particles should be resistant to DNase treatment through their protective protein coat such as a capsid and/or envelope, but some DNA viruses may be sensitive to DNase treatments. Studies have shown decreasing viral particles between samples with and without DNase treatment (Bettarel et al., 2000; Briese et al., 2015). Future studies could incorporate the use of biotinylated oligonucleotide probes (targeted viral enrichment) to capture viruses from complex matrices after sequencing library preparation to avoid upstream enzymatic treatments (Briese et al., 2015; Martínez-Puchol et al., 2020, 2022; Bonny et al., 2021). Although several studies use filtration (Allen et al., 2011; Fontenele et al., 2019; Garcia-Heredia et al., 2021; Patterson et al., 2021; Crum et al., 2023; LaRocca et al., 2023) to reduce background host and bacterial nucleic acids, it too can cause losses of viruses in filtration.

Although the third generation nanopore sequencing produces longer sequencing reads, it can be prone to higher error rates and low-quality scores, compared to short-read sequencing. The use of polishing after *de novo* assembly for error correction of substitutions, insertions and deletions has proved to increase assembly accuracy (Huang et al., 2021; Lee et al., 2021; Liu et al., 2022). Medaka polisher has been shown to decrease error rates after assemblies with MetaFlye or Canu (Goldsmith et al., 2020, 2021; Brancaccio et al., 2021; Wick et al., 2021). Future studies could include different combinations or rounds of polishing with Medaka or a combination of multiple types of polishers to enhance genome assembly accuracy. Furthermore, deeper long-read nanopore sequencing flow-cells such as the ONT PromethION (Yahara et al., 2021) with the updated Dorado^4^ (Pugh, 2023) base-caller and flow-cell chemistries that can reach quality scores of ~20 could be used in future studies to increase sequencing reads. In this study, different versions of the MinKNOW and Guppy basecaller software were used, due to frequent updates of the software packages by ONT. The different software updates would not affect the results of this study, because the same sequence files were used to compare Canu and MetaFlye assemblies. However, this may produce varying basecalling and/or read depth results when sequencing of viruses in environmental samples.

## 5. Conclusion

In this study, long-read metagenomics sequencing using a portable nanopore MinION sequencer allowed the detection of viruses in fecal and serum samples of marine mammals that are known to cause diseases in vertebrate and invertebrate hosts. This suggests that marine mammals could be used as sentinel species of ocean and human health by monitoring for emerging pathogens under a One Health framework. This sequencing method coupled with the sequence assembler, Canu identified *Parvoviridae, Annelloviridae* and *Circoviridae* that were confirmed with the NCBI viral protein (BLASTx) and nucleotide (BLASTn) Refseq databases. The data analysis approach presented here will be useful for virus surveillance using a long-read metagenomics sequencing.

## Data availability statement

The datasets presented in this study can be found in online repositories. The names of the repository/repositories and accession number(s) can be found at: NCBI—PRJNA998092.

## Ethics statement

Samples from Navy animals were collected during their routine care and under the authority codified in U.S. Code, Title 10, Section 7524. Secretary of Navy Instruction 3900.41H directs that Navy marine mammals be provided the highest quality of care. The U.S. Navy Marine Mammal Program (MMP), Naval Information Warfare Center (NIWC) Pacific, houses, and cares for a population of bottlenose dolphins and California sea lions in San Diego Bay (CA, United States). NIWC Pacific is accredited by The Association for Assessment and Accreditation of Laboratory Animal Care (AAALAC) International and adheres to the national standards of the U.S. Public Health Service policy on the Humane Care and Use of Laboratory Animals and the Animal Welfare Act. The study was conducted in accordance with the local legislation and institutional requirements.

## Author contributions

KV: conceptualization, methodology, validation, formal analysis, investigation, data curation, writing—original draft, visualization. TA: conceptualization, resources, writing—review and editing, supervision, project administration, funding acquisition. All authors contributed to the article and approved the submitted version.

## Funding

This research was funded by the Office of Naval Research (ONR), U.S. Department of the Navy under grant no. N00014-20-1-2117.

## Conflict of interest

The authors declare that the research was conducted in the absence of any commercial or financial relationships that could be construed as a potential conflict of interest.

## Publisher’s note

All claims expressed in this article are solely those of the authors and do not necessarily represent those of their affiliated organizations, or those of the publisher, the editors and the reviewers. Any product that may be evaluated in this article, or claim that may be made by its manufacturer, is not guaranteed or endorsed by the publisher.

## Footnotes

^1^ https://github.com/nanoporetech/medaka

^2^ https://github.com/marbl/canu

^3^ https://github.com/fenderglass/Flye

^4^ https://github.com/nanoporetech/dorado
